# Polar-coordinated contour processing algorithm in optimizing SCART treatment volume

**DOI:** 10.3389/fonc.2026.1720808

**Published:** 2026-05-14

**Authors:** Junqi Song, Japan Patel, Weihua Qi, Xuebing Shi, Yazhi Wang, Matthew Schmidt, Jun Yang, Dawei Wu

**Affiliations:** 1 State Key Laboratory of Mechanics and Control for Mechanical Structures, Nanjing University of Aeronautics and Astronautics, Nanjing, Jiangsu, China; 2 Gateway Scripts LLC, Saint Louis, MO, United States; 3 Precision Cancer Center, Foshan Fosun Chancheng Hospital, Foshan, China; 4 Precision Cancer Center, Nanjing Jiangbei Hospital, Nanjing, Jiangsu, China; 5 Precision Cancer Center, Quanzhou Binhai Hospital, Quanzhou, Fujian, China; 6 Foshan Fosun Chancheng Hospital, Washington University in St Louis School of Medicine, St. Louis, MO, United States

**Keywords:** bulky tumor, contour processing algorithm, SCART, script, SFRT

## Abstract

Spatially Fractionated Radiation Therapy (SFRT) has emerged as an appealing approach in radiation oncology due to its outstanding performance in tumor control and ability to preserve normal tissues. With the introduction of Stereotactic Centralized Ablative Radiation Therapy (SCART) as a new approach of SFRT techniques ablating the core of bulky tumor, a notable number of patients have been treated globally. However, SCART Treatment Volumes (STVs) are usually manually generated by physicist based on GTV volume using the contour editing tool of the treatment planning systems (TPS). It is not only tedious but also suboptimal in terms of desired position, shape and volume. To overcome these challenges, our group proposed a creative algorithm based on polar-coordinated calculations principle to help physicist generate the STV. This approach has achieved several improvements, reducing time by approximately 90% and minimizing the need for recontouring. In addition, the STVs demonstrated a 15% increase in volume, as well as an enhanced conformality comparing with manual contour. This method has improved both efficiency and reproducibility of SCART planning with optimal STV.

## Introduction

Spatially Fractionated Radiation Therapy (SFRT) is a unique radiation philosophy by intentionally delivering a heterogeneous, ablative dose to part of the tumor volume in a few (1~3) fractions. By interspersing high-dose peaks and low-dose valleys inside the gross tumor volume (GTV), SFRT destroys part of the tumor volume in targets while reducing toxicity to adjacent normal tissues. This non-uniform dosing philosophy is particularly valuable for bulky or radio-resistant tumors, where conventional or SBRT irradiation can exceed normal tissue tolerances (1). Beyond direct toxicity at peak regions, SFRT induces bystander and abscopal effects mediated by cytokine release and immune activation from irradiated valley areas. Preclinical microbeam studies also highlight a transient “permeability window” in tumor vasculature post‐peak exposure, enhancing drug delivery and potential synergy with chemotherapeutics or immunomodulator (2).

Despite its promise, SFRT faces significant limitations. A major challenge is the lack of standardization: institutions employ highly variable peak-valley geometries, peak‐dose prescriptions, and fractionation schemes, which weakens reproducibility and slows the development of standard protocol. Furthermore, the inherent technical complexity, especially with 3D Lattice, demands advanced treatment planning strategies, complex quality‐assurance procedures, thus further slows down the process of broader adoption. These difficulties reveal the urgent need for standardized guidelines and fluence workflows to facilitate consistent implementation and robust evaluation of SFRT across treatment centers (3).

## Lists of SFRT approaches

### First-generation GRID therapy

The earliest form of Spatially Fractionated Radiation Therapy (SFRT), called GRID therapy, originated in the kilovoltage x-ray era. To treat large or deep tumors without intolerable skin toxicity, physicians used perforated copper or lead screens (physical grids) between the x-ray source and the patient. Shielded skin regions acted as healthy “seeds” for later regeneration. Typical grids had holes 1–2 cm in diameter with 2–4 cm spacing, producing peak doses 2–3 times higher than valley doses. Kohler (4) first applied this “perforated screen” method, and Liberson (5) reported successful treatment of deep cancers using it (6).

### Modern IMRT/VMAT-based GRID

Advances in linear accelerators, MLCs, and inverse planning enabled IMRT/VMAT-based GRID therapy, eliminating the need for physical blocks. Computerized optimization allows efficient, reproducible treatments for deep or complex tumors. Retrospective studies show that single-fraction GRID (15–20 Gy) provides symptom relief in 70–80% of patients and achieves pathologic complete responses up to 80%, with toxicity similar to conventional radiotherapy (7).

### Three-dimensional LATTICE therapy

In the 2010s, LATTICE therapy was introduced as second-generation, 3D form of SFRT (8). Unlike planar GRID, LATTICE delivers ultra-high dose “vertices” directly inside the gross tumor volume, creating internal peak–valley patterns while sparing normal tissues. Using VMAT or stereotactic techniques, spherical vertices (0.5–1.5 cm) are placed at regular intervals within the tumor, each receiving ablative doses (8–15 Gy per fraction), while surrounding tissue receives conventional doses (9). This volumetric design allows precise control of vertex geometry, spacing, and dose, reducing toxicity to normal tissues and organs at risk.

### Stereotactic centralized ablative radiation therapy

Stereotactic Centralized Ablative Radiation Therapy (SCART) represents the latest evolution of SFRT (10), combining the spatial modulation of GRID and LATTICE with the precision of stereotactic body radiation therapy (SBRT) (Yang, Jun, et al). In SCART, one single high-dose core region called SCART-TV (STV) is concentrated centrally within the GTV, providing a simple solution for SFRT. STV but not GTV would be prescribed and targeted to ablative doses (15–24 Gy per fraction in our practice), enables sharpest dose falloff, while maintaining a strict low-dose restriction of approximately 5 Gy at the GTV periphery. This non-uniform distribution exploits tumor heterogeneity to selectively intensify treatment in radioresistant zones.

In the first-in-human phase I dose-escalation study, 21 patients with bulky or metastatic tumors received escalating SCART regimens (15 Gy×1 to 24 Gy×3) with the border dose of 5Gy each fraction. No dose-limiting toxicities or grade III+ adverse events were observed, and median tumor volume reduction at first follow-up was 49.5% (SD 40.9, p = 0.009). Subsequent analyses report local control rates of 95%, with 14 of 21 tumors achieving partial or complete responses (11).

### STV generation

The SCART−Treatment Volume (STV) is contoured as a spindle−shaped sub-volume at the geometric center of the gross tumor volume (GTV), with its dimensions scaled proportionally to the overall GTV size based on the prescribed ablative (15–24 Gy) and border (5 Gy) doses;

It is found that the optimal axial dimension of STV is proportional to GTV and the proportion is recommended by Yang’s group.

$STV^{\prime }s\;Dimension=GTV^{\prime }s\;Dimension\times \frac{Protection\;Dose}{SCART\;Abliation\;Dose}$ (see **Table 1**).

**Table 1:** Recommended SCART shrink percentage by Dr. Jun Yang.

SCART dose	GTV border dose	DGTV/DSTV	STV diameter / GTV diameter	VSTV/VGTV
15Gy	5Gy	33%	36%	10.60%
18Gy	5Gy	28%	27%	6.30%
21Gy	5Gy	24%	24%	4.50%
24Gy	5Gy	21%	21%	3.30%

This spatial segmentation concentrates the highest radiation burden within the tumor’s hypoxic, treatment−resistant core. By focusing ablative doses on this central STV, SCART maximizes direct cytotoxicity and elicits robust bystander and abscopal immune effects, while the lower dose peripheral ring preserves adjacent normal tissue integrity.

Treatment fields are delivered using VMAT such that all beamlets intersect exclusively within the STV, thereby avoiding direct irradiation of the peripheral GTV. By limiting the maximum field aperture to the STV and employing two coplanar photon arcs, LINAC manages to achieve an exceptionally sharp dose fall−off at the STV boundary, generating a maximal dose gradient between the high−dose core and low−dose rim and optimizing tumor ablation alongside normal tissue sparing (11).

## Method

### Margin tool for SCART treatment volume generation

Varian systems offer multiple contouring utilities, among which the Margin tool is traditionally used for SCART Treatment Volume (STV) creation. This functionality automatically generates an inner volume by applying a uniform shrinkage (or expansion) margin to the gross tumor volume (GTV), defining the STV for high-dose targeting. While straightforward and user-friendly, the Inner Margin tool’s isotropic margin application lacks direction-specific flexibility, limiting the customization of STV geometry in cases where non-uniform adaptation to adjacent critical structures or anatomical heterogeneities is desirable.

To utilize this tool, medical physicists must manually measure the maximum GTV diameter and calculate an appropriate shrinkage informed by both tumor volume and experiential judgment. This manual STV generation process typically involves iterative adjustment of the isotropic shrinkage in each axial, sagittal, and coronal direction—often consuming at least 30 minutes per case. Even seasoned practitioners rarely achieve optimal STV parameters on the first attempt, necessitating multiple refinements. When the manual method is used for STV generation, the resulting geometry is not always optimal. Consequently, during the subsequent plan optimization phase, peak dose leakage may occur. requiring the STV to be manually adjusted and re-contoured several times to achieve a suitable geometry for treatment planning.

A critical drawback of fixed-distance shrinkage is its insensitivity to variations in GTV cross-sectional diameters: the tool uniformly reduces the shrinkage across all slices, which can yield suboptimal STV geometries when directional adaptation to tumor shape and nearby critical structures is required.

**Figure 1:**
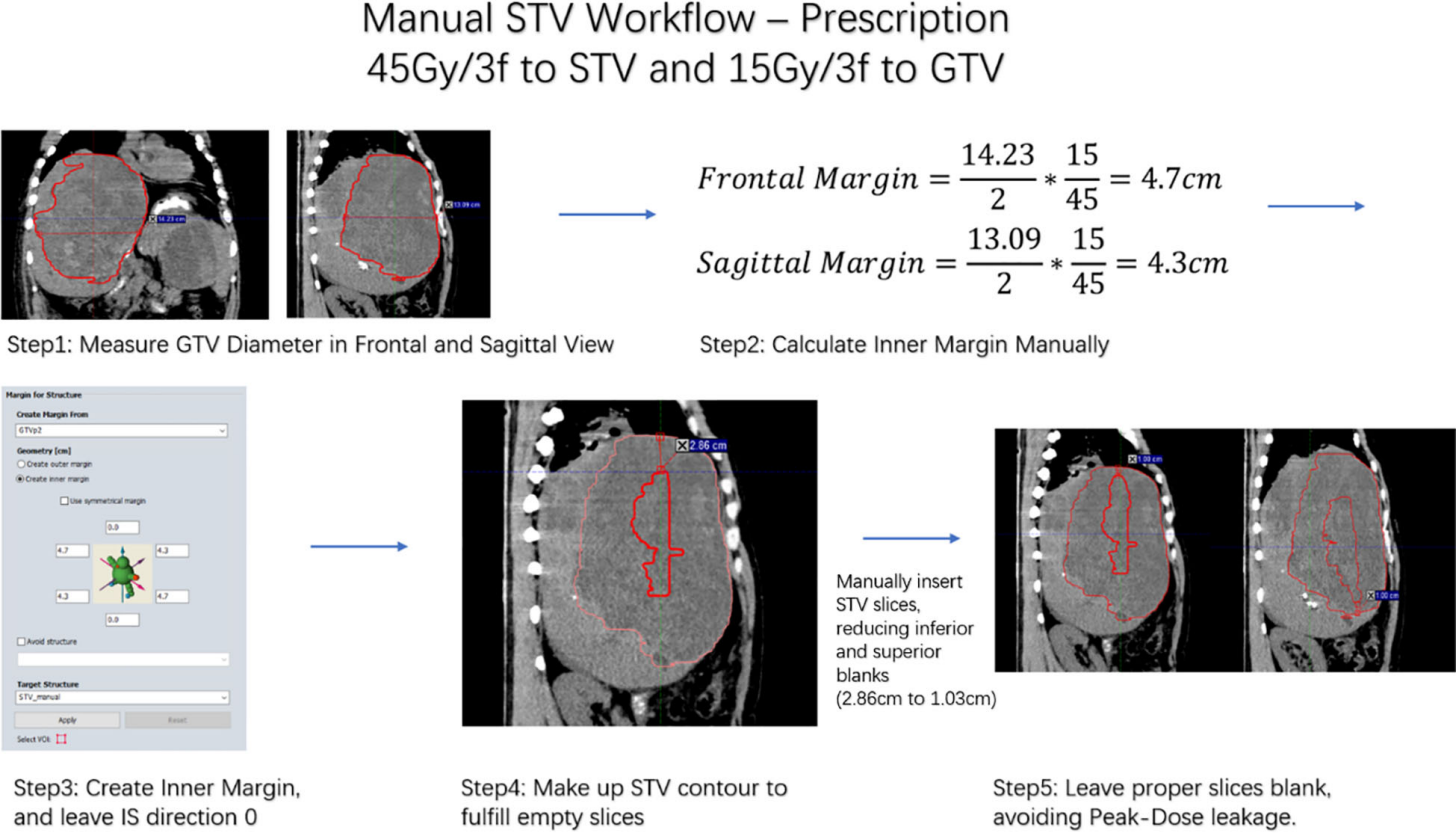
Workflow for manually STV generation.

Example workflow is shown in **Figure 1**.

### Polar-coordinated algorithm for STV generation - embedded in script

Based on Yang’s clinical research and experience with SFRT, a polar-coordinate method was developed to generate SCART Treatment Volume (STV) more efficiently. The algorithm sets shrinkage according to polar directions from the tumor center, enabling adaptive contouring that matches tumor shape while protecting nearby critical organs. **Table 1** shows Yang’s recommended shrinkage values, providing a standardized and practical approach for STV creation in different tumor geometries.

Dose‐escalation analysis within the SCART framework (**Table 1**) shows that the STV radius decreases as the prescription dose increases. To keep a fixed proportion of the GTV radius, the STV is set to 0.36, 0.27, 0.24, and 0.21 of the GTV radius for 15, 18, 21, and 24 Gy prescriptions, respectively. This approach preserves the peripheral 5 Gy limit while maximizing the central high dose.

Using a polar‐coordinate algorithm ensures that this proportionality is maintained in all directions. By sampling dose evenly around the tumor center, it avoids the gaps and anisotropies that can occur with Cartesian grid methods, especially in irregular tumors. In addition, any STV slice with a radius smaller than 2 cm is automatically removed to prevent high-dose leakage caused by insufficient dose fall-off in very narrow regions.

Normally, a **5–15 mm** gap is left at both the superior and inferior ends of the STV within the PTV (GTV) to accommodate tumor motion along the inferior–superior (IS) direction. This gap helps ensure that high-dose regions remain confined to the intended tumor volume despite respiratory or positional shifts, improving treatment safety and robustness. As a result, polar‐coordinate planning maintains radial symmetry of the STV and also improves both computational speed and dose accuracy compared to traditional Cartesian approaches (12).

**Figure 2:**
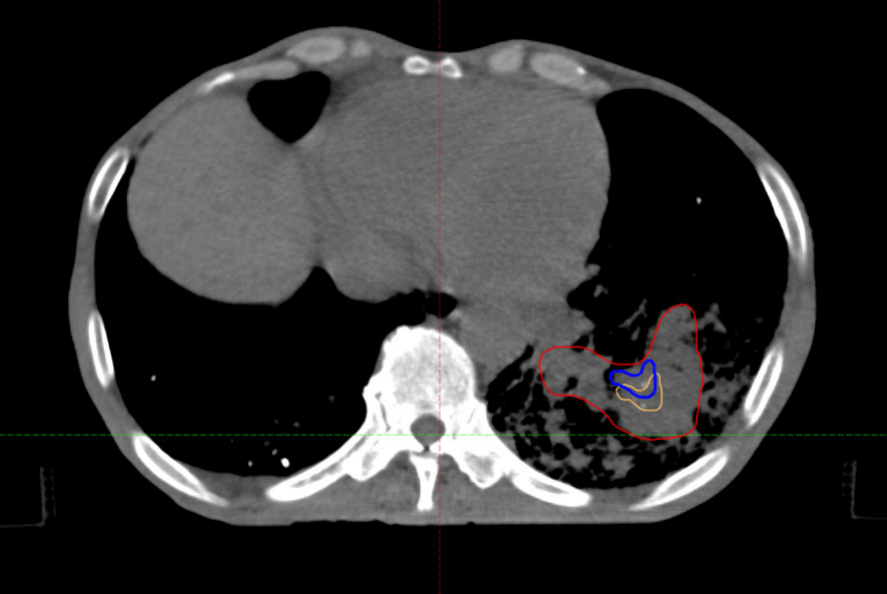
Demonstration of STV Generation with and without center shifting. (blue as non-shift, yellow as shifted, red as original GTV).

Shifting the graphical center is essential in SCART planning because tumors are irregular and asymmetric. The geometric centroid of a tumor often does not match its true center of mass. If the graphical center is not adjusted, the gap between the STV and GTV may be miscalculated. This can cause the distance between STV and GTV boundry to be too small, risking peak-dose leakage to normal tissue, or too large, reducing tumor coverage. Such misalignment would compromise treatment accuracy and safety, requiring manual corrections that are less efficient and more error-prone. By shifting the graphical center, the algorithm ensures proper alignment, improves reliability, and makes the method clinically practical. (See **Figure 2**).

The shifting algorithm starts by calculating the two-dimensional centroid of the GTV contour in the transverse plane as an initial reference point. Each contour coordinate is then expressed in polar form—defined by angle and distance from the centroid.

A refinement loop then adjusts this centroid to better approximate the tumor’s true center of mass. In each step, the algorithm compares the longest and shortest radial distances and shifts the centroid slightly toward the direction of maximum distance. This process repeats until the centroid stabilizes, ensuring the STV is symmetrically centered within the GTV.

With these steps—centroid calculation, polar transformation, iterative realignment, and radial constraint—the automated script generates a smooth, well-centered STV contour in under one minute. This automation applies exclusively to STV contour generation and does not include treatment planning or dose optimization, which remain separate processes performed by the physicist using the TPS.

Importantly, this algorithm operates exclusively on contour structures and does not rely on diagnostic or simulation CT image intensity information. This method avoids variability introduced by imaging quality, contrast protocols, or scanner-specific parameters, thereby improving robustness and cross-platform compatibility.

This structure-only design also facilitates seamless integration into treatment planning scripts for automated STV contour generation. The algorithm only produces the STV contour structure. Physicists then use these contours for subsequent treatment planning and optimization. This design makes the algorithm applicable across different institutions and imaging protocols without modification.

**Figure 3:**
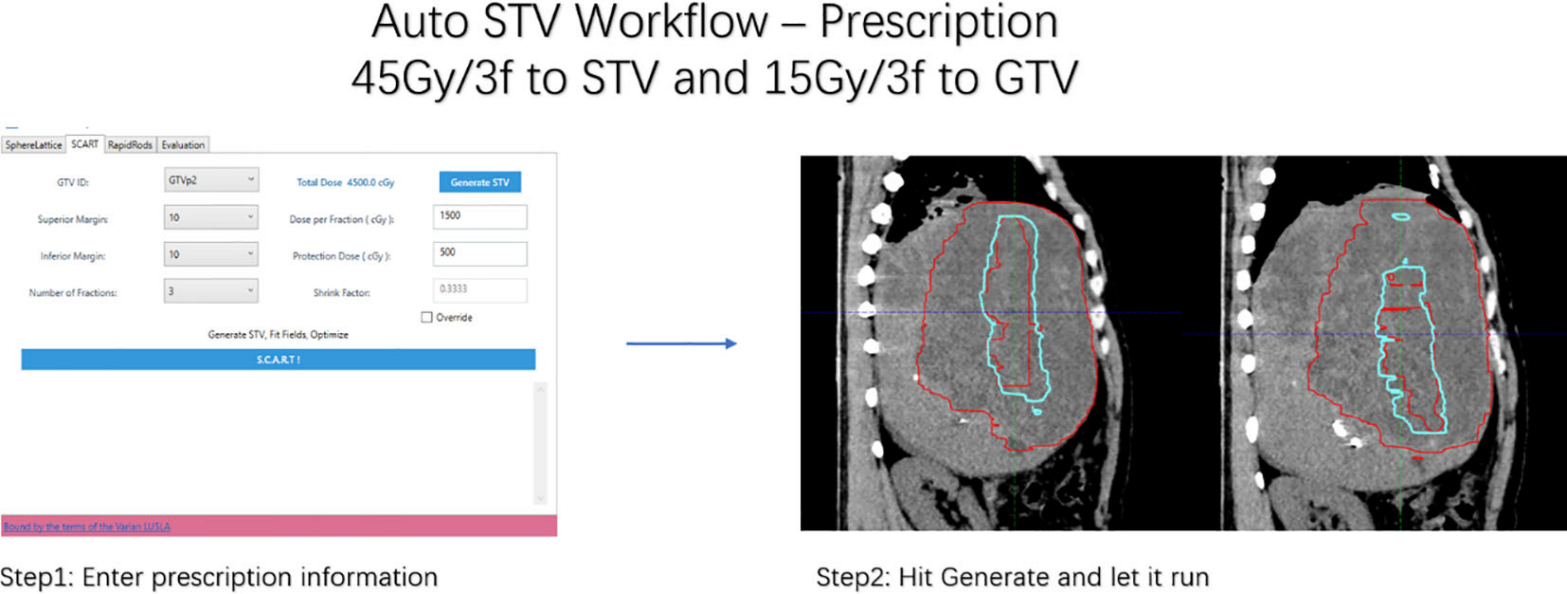
Workflow for polar-coordinated auto STV generation, together with STV comparation. (red as manual and cyan as automated).

Workflow is shown in **Figure 3**.

### Clinical strategy

Following STV generation, during the subsequent plan optimization phase, protection of organs at risk (OARs) is assigned higher priority than preservation of the nominal STV volume. A 3mm control ring structure surrounding the planning target volume (PTV), defined as equivalent to the GTV with zero margin, is implemented to enforce peripheral dose limitation. This control ring is constrained to a maximum dose of 5 Gy per fraction, ensuring steep dose falloff from the central high-dose region. In addition, the STV is required to maintain a minimum separation distance of 1.5 cm from critical OARs. When conflicts arise between STV geometry and OAR constraints, the STV volume is adaptively reduced or reshaped to satisfy OAR dose limits, reflecting the clinical priority of normal tissue safety over central dose escalation.

### Limitation

This algorithm is specifically designed for co-planar photon beam delivery systems and assumes a rotationally symmetric dose distribution achievable under co-planar irradiation conditions. It is not intended for proton therapy or other particle-based treatment modalities, where fundamentally different dose characteristics invalidate the underlying geometric assumptions. For non-co-planar photon delivery techniques—such as HyperArc or CyberKnife—the algorithm can still be executed; however, it is likely to underestimate the effective STV volume due to enhanced angular coverage and increased dose conformality inherent to these systems. Consequently, direct application of this method in non-co-planar settings may result in overly conservative STV definitions and should be approached with caution or supplemented by modality-specific adjustments.

## Results

A direct comparison of manually generated STVs and algorithm-generated STVs (**Table 2**) highlights key performance improvements achieved with the automated method. This analysis was conducted using the same cohort of 21 patients with bulky liver tumors; each patient had two STVs generated (manual and automated) for comparison. In clinical practice, a prescription dose of 54 Gy in 3 fractions was delivered to the STV, while 15 Gy in 3 fractions was prescribed to the PTV (defined as the GTV with zero margin). Across all treatment plans, Dmax to all organs at risk remained below 15 Gy delivered in 3 fractions. Given that these doses were substantially lower than established tolerance thresholds, detailed organ-specific dosimetry report was not considered necessary and is therefore not presented.

**Table 2:** Data comparison for manually and automate generated STV of 21 bulky tumor patients.

Dose region (cGy)								
GTV Volume (cc)	[1500, 3000]		[3000, 4500]		[4500, 5400]		> 5400	
	Manual	Auto	Manual	Auto	Manual	Auto	Manual	Auto
2136.8	59.9	74.06	15.4	12.86	6.9	3.87	9.1	8.5
909.7	67.4	72.8	12.9	13.5	4.7	4.98	7.8	7.43
876.6	52.1	61.05	12.8	11.57	6	3.72	6.1	8.12
1082.2	44.3	58.02	9.3	11.69	3.5	4.07	4.7	6.56
125.6	57.8	61.15	17.2	13.46	8.3	4.7	6.2	8.04
183.6	62.6	69.17	17.2	14.38	9.6	4.85	6.3	8.99
961.5	57.5	61.56	11.3	11.88	4.6	4.24	5.8	7.33
637.6	59.1	63.68	14.8	12.26	5.8	4.27	8.3	7.75
373.9	52.1	59.29	11.7	11.69	4.5	3.85	7.3	8.1
1837.4	52.9	63.25	9.6	11.56	4.4	4.27	4.6	7.11
1155	44.5	63.01	10.3	11.98	3.8	4.23	6.5	7.75
961.4	64.5	60.77	19	11.86	6.3	3.98	9.5	7.66
1587.8	70.2	73.28	14.4	13.07	5.3	3.93	7.9	8.57
2370.3	46.1	61.94	10.1	11.66	5	3.92	4.7	7.28
312.6	68.4	62.83	13.8	12.83	4.7	4.22	7.5	8.48
218.6	61.5	64.36	13.1	14.27	4	4.99	7.2	7.64
928.2	54.9	61.95	9.3	11.85	3.2	4.07	5.2	7.4
1979.6	58.5	60.79	12.6	11.88	5.1	4.3	6.3	6.8
643.6	69.5	70.96	15.2	13.25	6.2	4.24	7.3	8.86
319.6	62.1	59.51	16.5	12.98	7.1	5.04	5.3	7.63
157	62.4	61.66	17	13.69	6.2	4.78	9.2	7.9
Average	58.5	64.1	13.5	12.6	5.5	4.3	6.8	7.8
STD V	7.58	4.78	2.85	0.9	1.55	0.4	1.46	0.62

In manual STV creation, the GTV volume distribution across dose regions demonstrated considerable patient-to-patient variability. The low-dose (1500–3000 cGy) and high-dose (>5400 cGy) regions showed broad spreads in percentage volumes, reflecting inconsistent contouring and potential reproducibility challenges.

In contrast, the automated algorithm yielded a more uniform distribution of GTV volume across dose bands. Notably, the low-dose band (1500–3000 cGy) averaged 64.05% of the GTV with a standard deviation of 4.78%, and the next band (3000–4500 cGy) averaged 12.58% with a standard deviation of just 0.90%. The highest dose band (>5400 cGy) comprised 7.80% on average, higher than in the manual process, indicating that a larger proportion of the tumor received ablative doses. These lower standard deviations and higher high-dose volume indicate enhanced consistency, improved plan quality, and improved dose distribution consistency.

**Figure 4:**
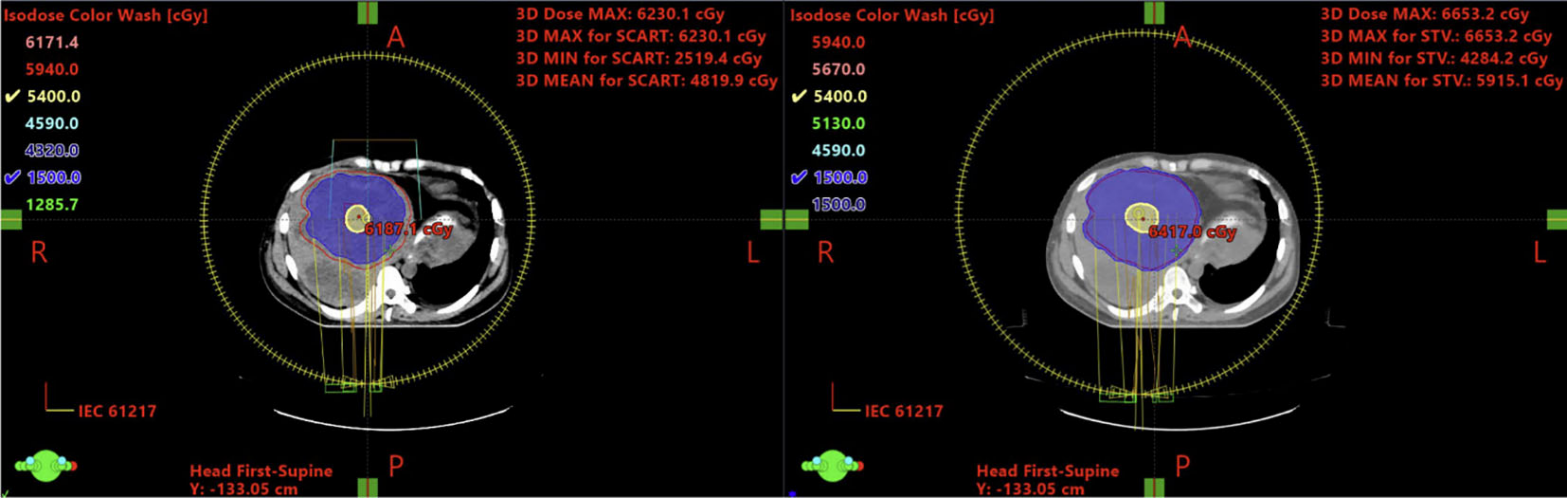
Left is manual plan, a smaller STV is generated, which influences both STV% over GTV, as well as the coverage of boundary dose (5Gy/fx). Compared with the plan on the right, which generated by script, having a bigger STV volume and smoother boundary dose.

A direct visual comparison of dose distribution in transversal view is provided in **Figure 4**.

**Figure 5:**
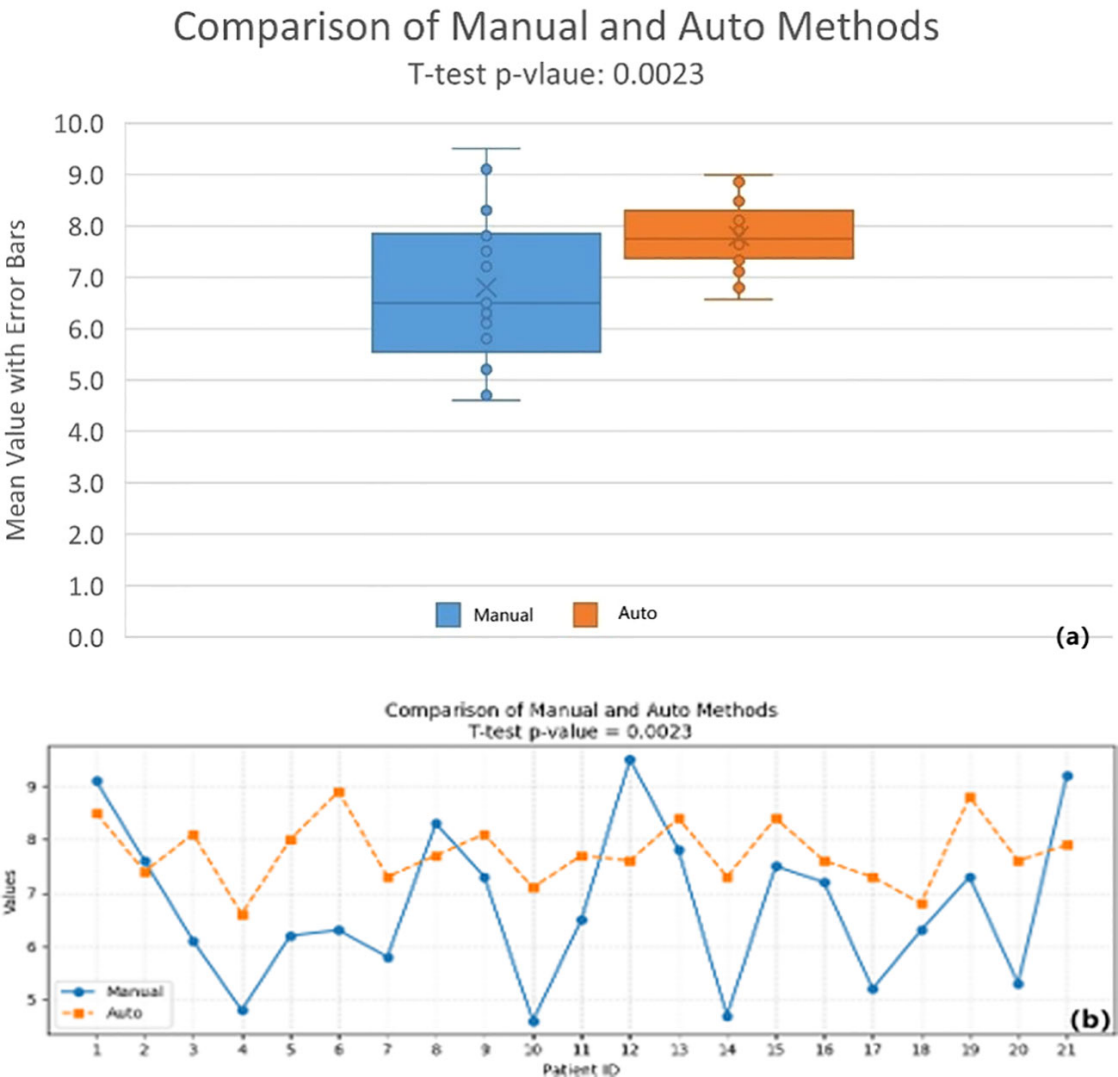
**(a)** Box plot comparison of manual and auto-generated STV volume with error bars. **(b)** Line chart comparison of manual and auto-generated STV volume.

To quantify the reproducibility and quality improvements afforded by the automated algorithm, we performed a paired comparison of key dose-volume metrics between manual and automated STV approaches for the same patient cohort. The two-tailed p-value was 0.0023, indicating that the observed differences are highly statistically significant by conventional criteria (**Figures 5a, b**). This result confirms that the automated STV script provides reliable and consistent and reproducible plan metrics compared to manual techniques, with minimal uncertainty around the estimated effect size.

Overall, automating STV generation reduces variability in critical dose regions, maximizes high-dose coverage, and ensures reproducible plan metrics, key factors for robust clinical adoption of SCART protocols.

## Conclusion

This study underscores the substantial advantages of a code-driven, polar-coordinated SCART STV generation over conventional manual workflows. First, automated STV creation delivered markedly more consistent and reproducible dose distributions: manual methods exhibited wide variability in intermediate and high-dose regions, whereas the algorithm produced uniform coverage with lower standard deviations. Second, the algorithm increased high-dose (>5400 cGy) volume coverage compared to manual planning, an important factor for optimizing tumor control, suggesting potential for improved dose delivery consistency.

Beyond dosimetry benefits, the polar-coordinated approach dramatically accelerates the STV generation step. Manual STV contouring required approximately 15 minutes and often necessitated multiple re-contouring attempts (5–6 on average) to achieve suitable geometry for planning, while the automated script completed STV generation in 1–2 minutes with only 1–2 attempts. Note that these time estimates refer specifically to STV contour generation and do not include the separate treatment planning and dose optimization process. This reduction in labor and re-plan frequency minimizes human error and frees clinical staff for other critical tasks.

Importantly, automated STV generation also enhances quality control and protocol standardization. By producing consistent dose metrics across patients, the method supports reliable SCART implementation, fosters clinician confidence, and simplifies the integration of SCART into routine practice. These workflow efficiencies and dosimetry improvements position the automated polar-coordinated algorithm as a useful tool that may facilitate broader adoption of SCART in radiation oncology.

## Data Availability

The original contributions presented in the study are included in the article/supplementary material. Further inquiries can be directed to the corresponding authors. GitHub - Varian-MedicalAffairsAppliedSolutions/MAAS-SFRThelper: Medical Affairs Applied Solutions ESAPI plugin tools to aid in the creation of structure patterns (spheres) or irregular stuctures (straight and angled rods) which can be evaluated or aid in the creation of treatment plans with the intention of not covering traditional PTVs homogeneously. · GitHub.
